# Posterior tibial tendon dysfunction by bone imprisonment

**DOI:** 10.11604/pamj.2016.24.218.9162

**Published:** 2016-07-12

**Authors:** Hassane Zejjari, Khalid Rachid

**Affiliations:** 1 Department of Trauma and Orthopaedic Surgery, Military Hospital Moulay Ismail, Meknès, Morroco

**Keywords:** Dysfunction, posterior tibial tendon, bone imprisonmen

## Image in medicine

The posterior tibial muscle is the main functional support of the plantar arch its dysfunction is the main cause of acquired flat foot. This is a 32 year old patient who consults for progressive pain of the inside of the ankle and right foot with a considerable decrease in its sporting and professional activity. Examination reveals a collapse of the plantar arch. The radiological assessment finds imprisonment of posterior tibial tendon in the internal retromalleolar bony canal. The patient received a release of the tendon with resection of the bony canal in full. The posterior tibial tendon showed longitudinal laceration was sutured and the internal retromalleolar canal was closed. The race and the freedom of the tendon were considered satisfactory by the end of surgery. The evolution was marked by the disappearance of pain, recovery of a satisfactory sport and professional activity and a progressive decrease in the collapse of the plantar arch.

**Figure 1:**
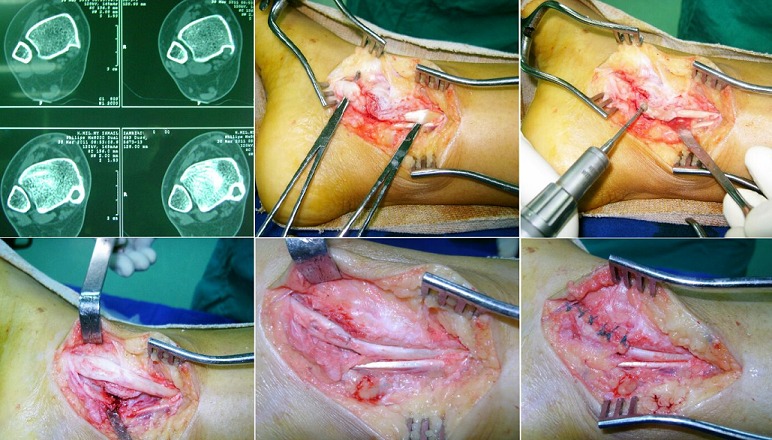
Appearance of bone imprisonment of posterior tibial tendon on a scanner of the ankle and the various stages of his surgical liberation

